# Clinical Features of Refractory Ascites in Outpatients

**DOI:** 10.6061/clinics/2017(07)03

**Published:** 2017-07

**Authors:** Wanda Regina Caly, Rodrigo Martins Abreu, Bernardo Bitelman, Flair José Carrilho, Suzane Kioko Ono

**Affiliations:** Departamento de Gastroenterologia, Divisao de Gastroenterologia e Hepatologia Clinica, Faculdade de Medicina FMUSP, Universidade de Sao Paulo, Sao Paulo, SP, BR

**Keywords:** Ascites, Hepatic Encephalopathy, Liver, Liver Cirrhosis, Outpatients

## Abstract

**OBJECTIVES:**

To present the clinical features and outcomes of outpatients who suffer from refractory ascites.

**METHODS:**

This prospective observational study consecutively enrolled patients with cirrhotic ascites who submitted to a clinical evaluation, a sodium restriction diet, biochemical blood tests, 24 hour urine tests and an ascitic fluid analysis. All patients received a multidisciplinary evaluation and diuretic treatment. Patients who did not respond to the diuretic treatment were controlled by therapeutic serial paracentesis, and a transjugular intrahepatic portosystemic shunt was indicated for patients who required therapeutic serial paracentesis up to twice a month.

**RESULTS:**

The most common etiology of cirrhosis in both groups was alcoholism [49 refractory (R) and 11 non-refractory ascites (NR)]. The majority of patients in the refractory group had Child-Pugh class B cirrhosis (*p*=0.034). The nutritional assessment showed protein-energy malnutrition in 81.6% of the patients in the R group and 35.5% of the patients in the NR group, while hepatic encephalopathy, hernia, spontaneous bacterial peritonitis, upper digestive hemorrhage and type 2 hepatorenal syndrome were present in 51%, 44.9%, 38.8%, 38.8% and 26.5% of the patients in the R group and 9.1%, 18.2%, 0%, 0% and 0% of the patients in the NR group, respectively (*p*=0.016, *p*=0.173, *p*=0.012, *p*=0.012, and *p*=0.100, respectively). Mortality occurred in 28.6% of the patients in the R group and in 9.1% of the patients in the NR group (*p*=0.262).

**CONCLUSION:**

Patients with refractory ascites were malnourished, suffered from hernias, had a high prevalence of complications and had a high postoperative death frequency, which was mostly due to infectious processes.

## INTRODUCTION

Ascites is one of the most frequent complications of liver cirrhosis and occurs in approximately 50% of patients with compensated liver disease during an observation period of approximately 10 years 1.

The development of ascites is associated with a worse disease prognosis, and the estimated average life expectancy is approximately two years for 50% of cirrhotic patients with ascites and only approximately six months for those with a urinary sodium excretion that is less than 10 mEq/L 2-4. This condition is observed particularly in cirrhotic patients with refractory ascites, which is thus indicative of the need for definitive treatment with a liver transplantation. According to the most-widely accepted pathophysiological hypothesis 5, ascites occurs only in the presence of portal hypertension, which leads to peripheral arterial vasodilatation and, consequently, effective arterial hypovolemia. Ascites triggers the activation of neurohumoral systems, the renin-angiotensin-aldosterone system, and the sympathetic nervous system (SNS); subsequently, ascites triggers the non-osmotic activation of antidiuretic hormone, which is a compensatory mechanism of organic homeostasis. These changes result in an increased retention of salt and water. During the later stages of cirrhosis, in addition to the maximal activation of neurohumoral systems, some patients will have an imbalance of the intrarenal regulatory hormones, leading to hepatorenal syndrome 6.

Depending on the duration of the underlying disease, this cascade of pathophysiological changes may lead to the development of ascites that can be easy or difficult to mobilize; thus, treatment is not restricted to the typical forms of therapy. In accordance with the most accepted pathophysiology, the typical treatment of ascites includes the restriction of salt in the diet and, in most cases, a combination of diuretics to obtain a satisfactory response, i.e., a negative balance of salt and water and the disappearance of the ascites and/or edema 7.

However, approximately 10% of patients with ascites will have no resolution even when they adhere to a low sodium diet (a maximum intake of 2.0 g salt/day, which is associated with the use of diuretics). This group includes patients who have an inability to mobilize ascites despite a confirmed adherence to the dietary sodium restriction (88 mEq salt/day) and the administration of the maximum doses of oral diuretics, such as 400 mg/day of spironolactone and 160 mg/day of furosemide (refractory resistant); these patients may experience a rapid re-accumulation of fluid after the therapeutic paracentesis despite their adherence to a sodium-restricted diet or develop diuretic-related complications, such as progressive azotemia, hepatic encephalopathy (HE), or progressive electrolyte imbalances, which require the discontinuation of the therapy (refractory intolerant) 8.

Thus, these patients are considered refractory to the conventional treatment of ascites and should be subjected to optional treatments, such as therapeutic serial paracentesis (TSP) with albumin replacement and intravenous insertion of a transjugular intrahepatic portosystemic shunt (TIPS); LeVeen or Denver valve insertions may be suitable for patients who are not eligible for TSP or TIPS. Moreover, a liver transplantation should be considered in the absence of contraindications 9,10.

Although this group of patients presents a poor clinical situation, in most cases, they do not reach a sufficient score on the Model for End-Stage Liver Disease (MELD) scoring system to justify a liver transplantation, which increases the risk of morbidity and mortality while awaiting definitive treatment 2,10.

Considering this scientific perspective, the objectives of this study were to present the clinical features and outcomes of outpatients who suffer from refractory ascites.

## METHODS

This observational study demonstrated our initial experience with such management at our institution and involved patients with cirrhotic ascites who were enrolled consecutively and prospectively and referred to the Clinic for Refractory Ascites of the Division of Gastroenterology and Hepatology – Hospital das Clínicas, University of São Paulo School of Medicine, São Paulo, Brazil, between March 2009 and August 2010. Patients were excluded if they were cirrhotic with ascites considered to have an easy resolution, ascites of a different etiology, and ascites with concomitant neoplasm or had current alcoholic hepatitis. Non-compliance with the dietary restrictions, drug therapy and ascites treatment guidelines was considered a cause of the false refractoriness.

Patients were included only after undergoing a comprehensive clinical evaluation to confirm the refractory ascites, which was based on their medical history, a physical examination and a plasma/ascitic fluid analysis (such as biochemistry, leucometry and culture). The patients participated in a one-week orientation, during which a nutritionist focused on the diuretic treatment withdrawal and the sodium restriction diet; the orientation was followed by 24 hour urine tests to quantify sodium and total proteins. Additional examinations were usually performed, such as a chest X-ray, an electrocardiogram, an echocardiogram, an abdominal Doppler ultrasound, an upper gastrointestinal endoscopy, and arterial blood gases. The total count number and differential leukocyte/mm^3^ ascites analyses were performed in all patients who were subjected to the various paracenteses.

All patients were referred to a clinical multidisciplinary evaluation with a cardiologist, nutritionist and psychologist. The nutritional assessment was performed with anthropometric parameters using the triceps skinfold (TSF) and mid-arm muscle circumference (MAMC) according to procedures performed in other studies 11,12. The TSF was measured in mm using an appropriate caliper that was placed parallel to the major axis of the non-dominant arm between the acromion and the olecranon. The arm circumference (AC) was measured in cm using a conventional measuring tape without a clearance space that was placed at the midpoint between the acromion and the olecranon on the non-dominant arm during normal posture and relaxation.

The MAMC was measured in cm and was calculated based on the AC. These parameters, including the estimation of the percentage of depletion, were compared with the normal range. The patients were then grouped into mild, moderate and severe malnutrition groups based on these comparisons 13. A nutritional assessment was performed, and dietary guidance regarding sodium restriction and individualized supplementation was provided as necessary.

The included patients then received the treatment protocol and were evaluated weekly for the volume of the ascites, body weight, renal function parameters, and electrolyte dosages. If necessary, changes to their prescriptions or other changes to the massive paracentesis with the intravenous albumin replacement (8 g/liter of ascites removed) were performed 13,14. In all outpatient care visits, the Child-Pugh, MELD and MELD-Na scores were updated. All patients were under hepatocellular carcinoma (HCC) surveillance with abdominal ultrasound and alpha-fetoprotein.

The ascites treatment protocol initiated therapy in combination with a diuretic schedule, which was supplemented with spironolactone (100 mg once daily) and furosemide (40 mg once daily) in patients who showed an inefficiency in weight loss after one week on the low sodium diet. Before the start of the diuretic therapy, an evaluation of the renal function parameters, urea, creatinine, and electrolytes, such as sodium and potassium was performed, and the dosage of sodium and potassium in the 24 hour urine test was assessed 15. Patients returned for weekly reevaluation to verify the effectiveness of the applied management and/or the appearance of complications due to the use of diuretics. In any of these events, the diuretic therapy had an optimized dosage until it reached the maximum allowed or until the administration was interrupted in the case of the onset of complications, indicating the failure of this treatment modality according to the criteria described for refractory ascites 7,16. Diuretic-resistant ascites was defined according to the International Ascites Club 7,8. In summary, at least one of the following criteria is fulfilled in the absence of therapy with a nonsteroidal anti-inflammatory drug:

- An inability to mobilize the ascites (manifested as minimal to no weight loss) despite the confirmed adherence to the dietary sodium restriction (88 mEq [2000 mg] per day) and the administration of the maximum tolerable doses of oral diuretics (400 mg of spironolactone and 160 mg of furosemide once daily);
- Rapid re-accumulation of fluid after therapeutic paracentesis despite adherence to a sodium-restricted diet; and
- The development of diuretic-related complications, such as progressive azotemia, HE, or progressive electrolyte imbalances.

Patients who were withdrawn from the conventional treatment of ascites protocol were followed-up every two weeks at most and were controlled by TSP with an intravenous albumin replacement. The insertion of TIPS was indicated for those patients who were controlled by TSP, required this procedure more than twice a month and did not present contraindications; even in patients with no urinary sodium excretion, performing paracentesis every two weeks controls the ascites 17. Currently, this form of therapy is considered a secondary option in the treatment of refractory ascites 7,9,18,19.

The patients with esophageal varices were treated according to the Consensus of Variceal Bleeding of the Brazilian Society of Hepatology 20 with an elastic bandage on the medium and/or thick caliber veins with previous bleeding or hematocystic spots, whose presence is associated with beta-blocker drug treatments. The average blood pressure of each patient, spontaneous bacterial peritonitis (SBP) background and previous or current renal failure were considered. In selected cases, the treatment was exclusively an endoscopic treatment of the varicose veins.

The follow-up was performed from the date on which the refractory ascites was diagnosed in each patient to the closure of the study date (August 2010). The evaluation period ranged from 3 to 17 months.

### Ethics

The study protocol was approved by the Ethics Committee of the Hospital das Clínicas, University of São Paulo School of Medicine and conformed with the ethical guidelines of the 1975 Declaration of Helsinki (6th revision, 2008). Because this is an observational study, the ethics committee waived the requirement of informed consent from each patient included in the study.

### Statistical Analysis

For the qualitative variables, comparisons between the two groups were performed using the chi-square test and Fisher’s exact test. This study adopted the 5% level of significance, and analysis of variance (ANOVA) was used for all statistical calculations.

## RESULTS

During the study period, 60 patients were enrolled as follows: 49 refractory ascites patients (R) and 11 non-refractory ascites patients (NR).

The most common etiology of the cirrhosis in both the refractory and non-refractory groups was alcoholism. The average age was 57.5±8.8 years in the R group and 50.3±8.1 years in the NR group (p=0.010), and males were predominant in both groups (72.9% in the R group and 66.7% in the NR group; p=0.712) (Table 1).

The nutritional assessment showed a protein-energy malnutrition in 81.6% of the patients in the R group and 35.5% of the patients in the NR group (Table 2). HE, hernia, SBP and hepatorenal syndrome (HRS) type 2 were present in 51%, 44.9%, 38.8% and 26.5%, respectively, of the patients in the refractory ascites group and 9.1%, 18.2%, 0% and 0%, respectively, of the patients in the non-refractory ascites group (*p*=0.016, *p*=0.173, *p*=0.012 and *p*=0.100, respectively).

Of the 49 refractory patients, only 5 underwent the insertion of TIPS. TIPS were inserted in patients who lost control of the ascites by paracentesis or if this procedure had to be performed twice a month without any absolute contraindications for TIPS. Although some patients needed three or more paracenteses per month, several criteria contraindicated the placement of TIPS. Although we had patients with MELD scores <18 and Child-Pugh Class B, some patients had persistent encephalopathies and/or grade >2, HRS type 2 and/or other changes to the procedure that contraindicated or would be controversial to its effectiveness. In total, 12 (24.49%) patients in the refractory group and 1 patient in the non-refractory group required hospitalization during the evaluation period. The causes of admission varied, including decompensated ascites preventing the insertion of TIPS (5), severe gastrointestinal bleeding (1), infection (3), and HE (3).

Five TIPS were inserted, but 4/5 (80%) patients presented HE and required diuretics to control the ascites; 3/5 (60%) patients who received TIPS required low doses of diuretics to control the ascites. Furthermore, despite the use of diuretics at tolerated doses, 1/5 (20%) patients returned to controlling the ascites by serial paracentesis because of TIPS obstruction 30 days after the insertion, and this patient presented with a worsening clinical condition and died on the 4^th^ month after the TIPS insertion due to an acute myocardial infarction.

Of the total number of enrolled patients, 6 patients (54.54%) in the non-refractory group and 20 patients (40.81%) in the refractory group received propranolol for more than 30 days as a primary or secondary bleeding prophylaxis from gastroesophageal varices.

Mortality occurred in 28.6% of the patients in the R group and 9.1% of patients in the NR group (*p*=0.262). The results of the clinical complications diagnosed in both groups are shown in Table 3. Of the 15 total deaths, 5 patients were post liver transplantation, and 10 patients were on the waiting list for transplantation. According to the total deaths, 14 were in the refractory group; 9/14 patients were on the liver transplantation list; 3/9 deaths were caused by umbilical hernia rupture; and 2/9 deaths were caused by SBP, one of which progressed to sepsis, and the other progressed to HRS. In the other four cases (4/9), 3/9 deaths were not directly related to the presence of the ascites (rather, they were related to acute arterial thrombosis of the lower limbs, upper gastrointestinal bleeding and pulmonary embolism), and the other died due to cachexia in the course of the refractory ascites with contraindications to liver transplantation, TIPS and paracentesis. Five of the 14 deaths occurred post liver transplantation, and three of these occurred due to infectious complications. During this study, there were no cases with HCC since it was an exclusion criterion. SBP occurred in two cases (4.08%) of refractory patients.

## DISCUSSION

The treatment of ascites in liver cirrhosis is typically performed by different medical specialists. However, professionals other than gastroenterologists and hepatologists can also perform the treatment procedures, including gastroenterological surgeons, nephrologists and infectologists. Different medical doctors follow these patients depending on their degree of familiarity with ascites treatment, and they play a key role in the ultimate goal of therapy. However, many professionals do not follow any established treatment protocol, which may lead to the emergence of several complications, such as HE and renal dysfunction, which are the most frequent complications 7.

Because many physicians note difficulties in managing massive ascites, we aimed to introduce a protocol that identifies outpatient cirrhotic patients with ascites that is considered difficult to manage (due to cirrhosis decompensation). The patients were followed-up with nutritional assessments to verify their adherence to the diet guidelines according to their individual needs because there is a direct relationship between malnutrition and increasing complications in cirrhotic patients with refractory ascites. Nutritional parameters, such as body mass index and percentage of ideal weight, have limitations in chronic liver disease mainly because of fluid retention, which is often exhibited by patients, including those without detectable ascites 21.

According to our results, 80% of the patients had some degree of malnutrition in the initial evaluation, which was frequently related to moderate or advanced cirrhosis. This was also the profile of our patients, and the majority of them were classified as Child-Pugh B 22,23; our results are consistent with results of other studies 24. In several studies, protein and calorie malnutrition have been associated with an increased number of serious complications, including ascites, variceal bleeding, increased surgical morbidity and mortality, and reduced survival 25,26. This fact is very important considering the results obtained in a recent study 27, which showed that better nutritional support for patients with refractory ascites led to lower rates of morbidity and mortality compared with patients with refractory ascites who did not have nutritional supplementation 23.

Thus, after excluding the other causes of ascites, which were not related to liver cirrhosis and/or associated with malignancies, the patients followed the treatment protocol for ascites and always paid attention to the exclusion of causes of false refractoriness 28-31.

Interestingly, among the complications presented during the treatment in our refractory patients, upper digestive hemorrhage (UDH), SBP, HE, HRS type 2, and umbilical and/or inguinal-scrotal hernia were the most prevalent complications and occurred in 38.8%, 38.8%, 51%, 26.5%, and 44.9% of the patients, respectively. According to the pathophysiology of ascites, the appearance of HRS type 2 is one of the expected complications in advanced stage cirrhotic patients with ascites, when the activation of the neurohumoral system, renin-angiotensin-aldosterone, SNS, and antidiuretic hormone (ADH) are extreme and can no longer return the balance of the hemodynamic parameters to normal levels of blood pressure.

Thus, in some patients, there will be an increased activation of vasodilator hormones or a moderate production of vasoconstrictors in the kidney, which often reduces the effect of systemic and renal vasoconstriction but elicits a renal failure that is stable for months. This renal failure is likely to develop into a more serious impairment, depending on external aggravating factors that are related to the presence of an acute circulatory dysfunction as it occurs, for example, during the onset of SBP 8, 21. During the advanced stage of cirrhosis, patients may not respond to the conventional treatment with diuretics, and several complications arising from its use can be diagnosed.

In addition, the patients frequently present asthenia, anorexia, malnutrition, hernias, HE, SBP and renal dysfunction. Thus, our results described findings that are related to severe cirrhotic patients with refractory ascites 22.

Although there were more patients with HRS and hernias in the refractory ascites group, no significant difference was observed in the frequency of these complications, which is likely due to the small number of patients included in the study. However, according to our results and the results of others, even in this condition, this group of patients did not achieve a MELD score that prioritizes liver transplantation, which is of main importance since this may have been related to the high percentage of deaths in the patients waiting for liver transplantation 23, 24. The MELD-Na appears to be a valid method to more readily benefit these patients 25, 26, 32.

We emphasize that refractory ascites patients are extremely and seriously ill (end-stage of liver disease). However, these patients do not need to reach a certain MELD score to be competitive with other cirrhotic patients on the liver transplantation list. In addition, only some patients obtain a score due to a special situation. Occasionally, the MELD score increases due to the absence of major coagulation disorders or a low alteration in the serum bilirubin level. Therefore, it is important to emphasize that the MELD-Na score could help hyponatremic patients with a worse prognosis, but it is not currently available. Thus, these patients progressively evolve with renal dysfunction and recurrent infections and may die before liver transplantation.

It is highly important to frequently diagnose the presence of hernias in the patients with abdominal distensions due to ascites since hernias were found to be a significant cause of death in the group waiting for liver transplantation. Complications of spontaneous perforations led to discussions regarding whether these patients would benefit from elective surgery for the hernias, particularly for those who presented with thinner and/or eroded skin 27, 33, 34.

Although five TIPS were inserted in the refractory patient group in accordance with the inclusion criteria for this type of therapy 9, 10, 18, we found that four of these patients developed HE, either grade 1 or 2, which was easily resolved and required diuretics at lower doses to control the ascites. We also emphasize that at the time of this study, the released TIPS were not covered, increasing the possibility of dysfunction and clinical complications 35. Several reports in the literature show the benefits of TIPS in controlling ascites compared to serial voluminous paracentesis 36-38. TIPS was performed in refractory ascites patients who underwent more than two paracenteses each month for ascites control. This frequency shows a loss of ascites control by paracentesis and highlights a poor life quality and malnutrition in this group of patients 39.

Our results are consistent with those reported in the literature; in five meta-analysis studies, the insertion of TIPS was shown to control ascites better than serial paracentesis therapies but leads to a higher incidence of HE 3, 40-44. This finding was highlighted in the EASL guidelines, which described that the reported management of ascites resulted more frequently in HE in the TIPS group compared to the paracentesis group 45. More recently, a retrospective study evaluating 70 covered TIPS and 80 serial paracenteses for refractory ascites treatment concluded that covered "stents" improved the survival to medium- and long-term without a significant increase in the short-term mortality of patients with refractory ascites in clinical treatment failure 46.

None of our clinical cases were consistent with post-paracentesis circulatory dysfunction, but this finding was not an aim of this study.

Regarding the frequency of deaths in our refractory patients, complicated hernias were the most frequent causes of death before transplantation, and bacterial infection was an important cause of death post liver transplantation, which was likely due to the advanced stage of the underlying disease and the degree of malnutrition 23, 47-49.

Among the refractory patients who died pre-transplantation, 4/9 (44.4%) were taking propranolol, and no cause of death was directly related to the refractory ascites; however, among the refractory deaths after transplantation, 2/5 (40%) had histories of propranolol use, and sepsis was the cause death. In the non-refractory group, one patient who received beta-blockers died due to SBP that progressed to sepsis. However, the sample presented was small.

In conclusion, patients with refractory ascites were very malnourished, frequently suffered from hernias and had a high prevalence of renal dysfunction, SBP, upper gastrointestinal hemorrhage, and episodes of HE. Moreover, when the patients reached a score eligible for liver transplantation, they had a high frequency of postoperative death mostly due to infectious processes. Thus, the developmental profile of refractory patients based on our results shows the necessity of reviewing the current criteria to prioritize liver transplantation and minimize the high morbidity and mortality in this group of patients. However, further studies with larger numbers of patients are needed to confirm these data.

## AUTHOR CONTRIBUTIONS

Caly WR and Ono SK conceived and designed the study. Caly WR, Abreu RM, Bitelman B, Carrilho FJ and Ono SK performed the study, analyzed the data, were responsible for the reagents/materials/analysis tools, provided clinical data regarding the patients and critical advice on writing the manucript. Abreu RM, Caly WR and Ono SK wrote the manuscript. Caly WR, Bitelman B, Carrilho FJ and Ono SK provided clinical data regarding the patients.

## Figures and Tables

**Table 1:** Clinical Results.

Parameters	R (49)	NR (11)	*p*-value
Age	57.5±8.8	49.9±7.9	0.010
Male gender (%)	73.5	63.6	0.712
Child-Pugh B (%)	71.4	36.4	0.034
MELD ± SD	13.4±4.2	11.2±4.0	0.126
MELD-Na ± SD	16.1±5.1	12.8±5.1	0.054

**Table 2:** Nutritional Status.

Nutritional Evaluation	R	NR	Total
Normal weight	4 (8.2%)	4 (36.4%)	8 (13.3%)
Mild malnutrition	7 (14.3%)	2 (18.2%)	9 (15.0%)
Moderate malnutrition	22 (44.9%)	2 (18.2%)	24 (40%)
Severe malnutrition	11 (22.4%)	1 (9.1%)	12 (20%)
Overweight	2 (4.1%)	1 (9.1%)	3 (5.0%)
Obese	3 (6.1%)	1 (9.1%)	4 (6.7%)

**Table 3:** Results of Clinical Complications.

Clinical complications	R (49)	NR (11)	*p*
HRS type 2	13 (26.5%)	0	0.100
HE	25 (51.0%)	1 (9.1%)	0.016
Hernias	22 (44.9%)	2 (18.2%)	0.173
SBP	19 (38.8%)	0	0.012
Hydrothorax	11 (22.4%)	1 (9.1%)	0.435
UDH	19 (38.8%)	0	0.012
Transplantation	9 (18.4%)	0	0.189
Mortality rate	14 (28.6%)	1 (9.1%)	0.262

Legend: HRS type 2: hepatorenal syndrome type 2; HE: hepatic encephalopathy; SBP: spontaneous bacterial peritonitis; UDH: upper digestive hemorrhage.
